# Age-related differences in muscle co-activation during locomotion and their relationship with gait speed: a pilot study

**DOI:** 10.1186/s12877-017-0417-4

**Published:** 2017-01-31

**Authors:** Hwang-Jae Lee, Won Hyuk Chang, Byung-Ok Choi, Gyu-Ha Ryu, Yun-Hee Kim

**Affiliations:** 1 Department of Physical and Rehabilitation Medicine, Center for Prevention and Rehabilitation, Heart Vascular and Stroke Institute, Samsung Medical Center, Sungkyunkwan University School of Medicine, Irwon-ro 81, Gangnam-gu, Seoul 135-710 South Korea; 2 Department of Neurology, Neuroscience Center, Samsung Medical Center, Samsung Medical Center, Sungkyunkwan University School of Medicine, Irwon-ro 81, Gangnam-gu, Seoul 135-710 South Korea; 3 Office of Biomedical Science, Research Center for Future Medicine, Samsung Medical Center, Samsung Medical Center, Sungkyunkwan University School of Medicine, Irwon-ro 81, Gangnam-gu, Seoul 135-710 Republic of Korea

**Keywords:** Aging, Co-activation, Electromyography, Gait speed, Stability, Locomotion

## Abstract

**Background:**

Muscle co-activation plays an important role in enhancing joint stability for movement regulation during motor learning activities. In normal aging, greater muscle co-activation is induced during gait in elderly adults. This study investigated age-related changes in muscle co-activation and spatio-temporal parameters during gait and identified the relationship between muscle co-activation and gait speed.

**Methods:**

A total of 46 adult volunteers participated in this study in three age groups (15 young adults [8 males, 7 females; age, 24.27 ± 2.71], 15 middle-aged adults [8 males, 7 females; age, 53.71 ± 2.52], and 16 elderly adults [7 males, 9 females; age, 76.88 ± 3.48]). All participants underwent locomotion analysis using a Three-dimensional motion analysis system and 12-channel dynamic electromyography.

**Results:**

The elderly adults showed significantly higher co-activation than the young and middle-aged adults during gait (*p* < 0.05). In contrast, elderly adults showed significantly lower trunk co-activation than the young and middle-aged adults (*p* < 0.05). Muscle co-activation was significantly correlated with gait speed by aging. Muscle co-activation of the trunk showed a significant positive correlation with gait speed based on age. However, muscle co-activation of the lower extremity showed a significant negative correlation with gait speed based on age.

**Conclusion:**

This finding demonstrated that less muscle co-activation of the trunk was related to locomotive instability in elderly adults. Therefore, clarification of the relationship between trunk co-activation and locomotor instability will be helpful for developing optimal rehabilitation of elderly people to prevent fall.

## Background

Normal aging causes a large change in walking ability. Elderly adults compensate for their reduced physical performance by being more cautious and by changing their gait pattern [1]. Compared to young adults, elderly adults’ gait pattern is characterized by slower gait speed, shorter step length, reduced range of motion at the hip, knee, and ankle joints, shorter relative swing phase, and a distal to proximal shift of joint torques [2, 3]. In particular, sarcopenia is a well described effect of aging, and a reduction in muscle strength and power is generally associated with loss of muscle mass [4]. It is also characterized by an overall decrease in the number of muscle fibers and cross-sectional area due to fatty and connective tissue replacement of the muscle fibers. This non-contractile tissue replacement may cause increased joint stiffness [5].

Muscle co-activation is the concurrent contraction of agonist and antagonist muscles that cross a joint. Muscle co-activation helps to enhance joint stability during movement regulation of motor learning activities [6]. Previous reports describe an increase in antagonist co-activation in the trunk and lower extremity muscles as a function of gait [7, 8]. In normal aging, greater muscle co-activation is induced during gait in elderly adults [7]. Increased muscle co-activation in elderly adults is most commonly described as a compensatory mechanism to increase joint stiffness that thereby enhances stability [9]. In addition, increased co-activation in elderly adults might be related with an increase in walking energy cost [7]. Despite this compensatory strategy, elderly adults still express an unstable gait pattern [10]. Consequently, co-activation of the trunk and lower extremity was important for maintaining gait stability in the elderly. Several studies have demonstrated that appropriate trunk co-activation promoted successfully dynamic balance and increased efficiency of gait [11, 12]. Age-related gait changes induce trunk instability, and elderly subjects who find it more difficult to control trunk stability during gait are at greater risk of falls [13]. In addition, other studies have shown that an increase in antagonist co-activation in lower extremity muscles leads to greater stabilization of gait [7, 14]. Hortobagyi et al. [7] showed that older adults exhibited approximately 83% higher co-activation in the thigh and shank. Lasen et al. [15] demonstrated that elderly adults exhibited 20% higher co-activation around the thigh during the stance phase when compared with young adults. However, aging related differences in muscle co-activation are based on a granular gait cycle.

Surface electromyographic analysis (sEMG) is used to estimate muscle activation and co-activation during gait. Assessment of trunk and lower extremity muscle activity can provide important information about muscular signals during gait [16]. Therefore, the purpose of this study was to clarify age-related differences in muscle co-activation of the trunk and lower extremity during gait among young, middle-aged and elderly adults. In addition, we investigated the relationship between muscle co-activation and gait speed by aging. We hypothesized that elderly adults had more co-activation of lower extremity muscles during gait than young and middle-aged adults. However, we also hypothesized that the elderly would have less co-activation of trunk muscle during gait than young and middle-aged adults. Finally, we hypothesized that muscle co-activation relates to gait speed by aging.

## Methods

### Participants

Fifteen young adults (8 males, 7 females; age, 24.27 ± 2.71), fifteen middle-aged adults (8 males, 7 females; age, 53.71 ± 2.52), and sixteen elderly adults (7 males, 9 females; age, 76.88 ± 3.48) participated in this study (Table 1). All subjects were right-side dominant. We performed the SPPB (Short Physical Performance Battery) assessment to evaluate physical functioning. Elderly participants in this study were considered high functioning if they scored ≥7 on the SPPB [17]. The participant characteristics are summarized Table 1. Prior to enrollment in the study, all participants provided written informed consent based on documents approved by the Samsung Medical Center Institutional Review Board.

**Table 1:** General characteristics of subjects

Parameters	Young	Middle-aged	Elderly
	(*n* = 16)	(*n* = 15)	(*n* = 16)
*General characteristics*			
Age (years)	24.27 ± 2.71	53.71 ± 2.52	76.88 ± 3.48
Sex (male/female)	8/7	8/7	7/9
Height (m)	166.53 ± 5.72	164.64 ± 7.95	160.75 ± 6.92
Body weight (kg)	59.53 ± 12.74	64.07 ± 11.38	59.72 ± 10.00
BMI (kg/m^2^)	21.76 ± 3.20	23.46 ± 2.51	23.59 ± 3.44
*Physical function level*			
SPPB	12.00 ± 0.00	11.93 ± 0.27	10.44 ± 1.15
*Cognitive function level*			
K-MoCA	29.91 ± 0.31	29.81 ± 0.51	25.01 ± 1.71
*medical histories (number of subjects)*			
Hypertension	0	4	9
Asthma	0	0	0
Diabetes	2	3	3
Osteoporosis	0	0	2
Arthritis	0	1	2
Arrhythmia	0	0	1

Values are expressed as the number or mean ± standard deviation

*BMI* Body mass index, *SPPB* Short Physical Performance Battery, *K-MoCA* Korean version of the Montreal Cognitive Assessment

### Experimental set-up

Gait parameters (gait speed, cadence, step length and step width) were collected at 120Hz using an infrared motion capture system with six cameras (Motion Analysis Corporation, Santa Rosa, CA, USA). The marker set used for this study is the Helen-Hayes model, which included 19 retroreflective markers (25 mm diameter) positioned on the pelvis and lower limbs. Six cameras were placed between 1–2 *m* and 6 *m* on a walkway in the gait laboratory. Muscle activation during gait was synchronously recorded using a 12-channel desktop direct transmission system sEMG device (Noraxon Inc., Scottsdale, AZ, USA). sEMG signals were collected at a sample frequency of 1000Hz, using 10 mm 3M^TM^ Ag/AgCl surface electrodes with an active area of 1 cm^2^ and an inter-electrode distance of 2 cm arranged in a bipolar configuration. The electrodes were positioned on the subjects’ right side on the rectus abdominis (RA), external oblique (EO), erector spinae of the lower back (ES), multifidus of the lower back (MF), gluteus maximus (GMAX), gluteus medius (GMED), rectus femoris (RF), vastus medialis oblique (VMO), adductor longus (ADD), biceps femoris (BF), tibialis anterior (TA), and the medial part of gastrocnemius (mGCM) muscles as described in the Surface Electromyography for the Non-invasive Assessment of Muscles project [18]. In addition, two foot switch sensors were placed on the plantar surface of each subject's foot in the right toe and heel positions. Each site was prepared by shaving, abrading, and cleaning the area with alcohol to reduce surface impedance.

### Experimental protocol

Before gait performance assessment, this study was normalized to amplitudes recorded during maximum voluntary contraction (MVC) of various muscles among the subjects. All the participants performed maximal back extension against manual resistance in prone positions for assessment of the ES and MF. In order to assess the RA and EO, subjects maximally flexed the muscles of their trunk, and manual resistance was applied to their extended arms and knees in supine positions with the knees bent to resist trunk rotation. The GMAX was assessed with maximal leg extension against manual resistance in a prone position. Subjects also performed maximal knee extension against manual resistance in sitting positions for assessment of the RF and VMO. The BF was assessed with maximal knee flexion against manual resistance in a sitting position. The ADD was assessed with maximal knee adduction against manual resistance in a sitting position. The TA was assessed with maximal ankle dorsiflexion against manual resistance in a sitting position. Finally, subjects undertook maximal plantar-flexion of their ankles against manual resistance in a standing position [19]. In all the assessments performed, MVC was maintained for ten seconds by all the subjects.

Participants were required to walk at a self-selected speed along a walkway 6 *m* in length. All participants wore their own athletic footwear for gait performance testing. Before formal measurements were started, practice sessions were performed to familiarize the participants with the procedure. Then, five trials were acquired per participant.

### Data analysis

The sEMG signals were sampled at 1000 Hz, and were band-pass filtered (10–350 Hz) and full-wave rectified with Noraxon software (MyoResearch XP Master Edition) [20]. This study processed the average normalized sEMG activity within selected phases of the gait cycle using MATLAB software (MathWorks, Inc., Natick, MA, USA) [21]. The selected phases of the gait cycle included the loading (0–10% of the gait cycle), mid-stance (10–30%), terminal stance and pre-swing (30–60%), initial swing (60–73%), and terminal swing (87–100%) phases [22]. The percent co-activation index (CI) is the percentage of co-activation between the agonist/antagonist muscles in the trunk part (RA:ES, RA:MF), the thigh part (RF:BF, VMO:BF, GMED:ADD), and the shank part (TA:mGCM), and was calculated using the following equation [14]:

$$ \mathrm{C}\mathrm{I}\ \left(\%\right)=2\left(\frac{{\displaystyle \int } min\left(sEM{G}_{agonist},\kern0.5em sEM{G}_{antagonist}\right)}{{\displaystyle \int }sEM{G}_{agonist}+{\displaystyle \int }sEM{G}_{antagonist}}\right)100 $$

### Statistical analysis

All data were analyzed using SPSS version 19 for Windows (SPSS Inc., Chicago, IL, USA). Statistical methods were used for the calculation of means and standard deviations. One-way ANOVA was used to compare the spatio-temporal data and co-activation data during gait between the young, middle-aged, and elderly subjects. Post-hoc analyses used Tukey’s honest significant difference tests. The relationships between gait speed and muscle co-activation during gait were assessed using Pearson’s correlation coefficient (r). Statistical significance was defined at *p* < 0.05.

## Results

### Spatio-temporal parameters

**Fig. 1:**
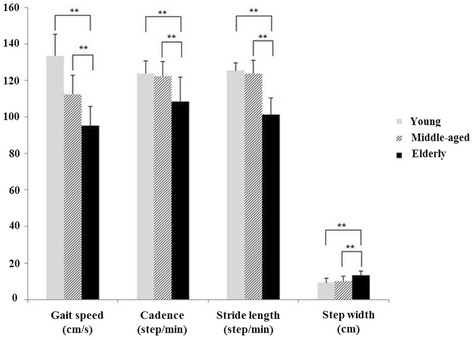
Age-related differences in spatio-temporal parameters during gait. Elderly adults exhibited slower gait speed, shorter cadence, shorter stride length, and larger step width than young and middle-aged adults (**p < 0.001)

There were significant differences in spatio-temporal parameters including cadence, stride length, and step width (*p* < 0.01) between the young, middle-aged, and elderly subjects. In Tukey’s HSD post hoc tests, the preferred gait speed of elderly adults was slower than the preferred gait speed of middle-aged and young adults. Elderly adults had shorter cadence and stride length than middle-aged and young adults. Finally, step width was larger in elderly adults than in middle-aged and young adults (Fig. 1).

### CI in stance phase

As illustrated in Table 2, in the initial contact phase, elderly adults had significantly decreased CI levels of the trunk part (RA:ES, *p* < 0.05; RA:MF, *p* < 0.01) by age. However, the thigh part of elderly adults showed higher CI levels (RF:BF, *p* < 0.05; VMO:BF, *p* < 0.05) than young adults. In the loading response and mid-stance phase, the trunk part of elderly adults showed a lower CI level (RA:ES, *p* < 0.05) than that of mid-aged and young adults. Elderly adults had significantly increased CI levels in the thigh part (VMO:BF, *p* < 0.05; GMED:ADD, *p* < 0.01) by age. In the terminal stance phase, only the thigh part (GMED:ADD) in the elderly had a significantly higher CI level than it did in young adults (*p* < 0.05). Finally, in the pre-swing phase, only the thigh part in elderly adults (RF:BF) had a significantly higher CI level than it did in young adults (*p* < 0.05). There were no significant differences in shank part CI based on age (Table 2).

**Table 2:** Muscle co-activation index (%) of mean normalized sEMG activity (%MVC) during gait

Gait cycle	Muscle pair	Young	Middle-aged	Elderly
		(*n* = 15)	(*n* = 15)	(*n* = 16)
*Stance phase (%)*				
Initial contact (0-2%)	RA:ES	20.32 ± 5.29	13.20 ± 8.62	7.40 ± 2.93^**†^
	RA:MF	23.71 ± 7.00	17.20 ± 5.24	4.69 ± 2.00^**††^
	RF:BF	17.39 ± 7.34	22.60 ± 8.17	27.72 ± 6.75^*^
	VMO:BF	16.73 ± 5.55	19.53 ± 7.54	24.45 ± 5.79^*^
	GMED:ADD	18.02 ± 3.34	20.63 ± 5.64	21.78 ± 4.90
	TA:mGCM	14.05 ± 6.42	14.44 ± 3.89	18.49 ± 7.50
Loading response (2-12%)	RA:ES	10.90 ± 5.81	3.65 ± 2.44	2.35 ± 1.51^*^
	RA:MF	7.19 ± 5.88	4.17 ± 2.14	2.24 ± 1.62^*^
	RF:BF	4.35 ± 1.47	4.76 ± 1.35	7.35 ± 3.28^*†^
	VMO:BF	3.27 ± 1.74	4.09 ± 1.15	5.76 ± 1.72^**†^
	GMED:ADD	4.17 ± 1.07	5.17 ± 1.34	6.44 ± 2.26
	TA:mGCM	3.97 ± 1.66	4.56 ± 1.36	4.99 ± 1.47
Mid-stance (12-31%)	RA:ES	3.65 ± 1.74	3.08 ± 1.63	1.52 ± 0.71^*†^
	RA:MF	3.44 ± 1.89	2.54 ± 1.76	1.66 ± 0.99^*^
	RF:BF	2.19 ± 1.62	2.87 ± 0.87	7.22 ± 1.06
	VMO:BF	1.83 ± 1.06	1.88 ± 0.71	2.65 ± 1.45
	GMED:ADD	1.43 ± 0.87	1.96 ± 0.77	4.67 ± 2.14^**††^
	TA:mGCM	1.77 ± 0.80	1.79 ± 0.82	1.90 ± 0.74
Terminal stance (31-50%)	RA:ES	1.57 ± 0.53	1.37 ± 0.68.	1.17 ± 0.70
	RA:MF	1.73 ± 0.90	1.71 ± 0.78	1.26 ± 0.34
	RF:BF	2.86 ± 1.38	2.85 ± 0.95	3.37 ± 2.27
	VMO:BF	1.94 ± 1.01	2.17 ± 0.79	2.70 ± 1.11
	GMED:ADD	1.65 ± 0.73	2.39 ± 0.67	2.90 ± 1.49^*^
	TA:mGCM	0.99 ± 0.18	1.10 ± 0.35	3.18 ± 0.50
Pre-swing (50-62%)	RA:ES	2.35 ± 1.39	2.32 ± 1.27	1.81 ± 1.31
	RA:MF	2.21 ± 1.24	2.01 ± 1.21	1.93 ± 1.17
	RF:BF	2.96 ± 1.33	3.07 ± 1.50	5.67 ± 2.27^*^
	VMO:BF	2.95 ± 1.57	2.88 ± 1.01	3.95 ± 1.16
	GMED:ADD	2.22 ± 1.03	3.36 ± 0.83	5.34 ± 1.44
	TA:mGCM	2.82 ± 1.06	2.99 ± 0.72	3.60 ± 1.41
*Swing phase (%)*				
Initial swing (62-75%)	RA:ES	3.28 ± 1.26	2.98 ± 0.96	1.03 ± 0.38^*^
	RA:MF	3.25 ± 1.45	2.98 ± 1.23	2.01 ± 0.34
	RF:BF	2.31 ± 1.28	2.61 ± 1.21	3.16 ± 0.97
	VMO:BF	1.87 ± 1.21	2.44 ± 1.48	2.87 ± 1.42
	GMED:ADD	1.47 ± 1.05	2.89 ± 1.15	2.98 ± 1.32
	TA:mGCM	2.42 ± 1.22	2.27 ± 1.08	1.69 ± 0.96
Mid-swing (75-87%)	RA:ES	5.91 ± 2.62	4.00 ± 1.03	1.86 ± 1.24^**†^
	RA:MF	5.32 ± 2.10	3.87 ± 1.81	2.21 ± 0.48^**†^
	RF:BF	3.04 ± 1.56	3.69 ± 1.21	5.82 ± 2.12^**^
	VMO:BF	2.50 ± 2.15	3.00 ± 1.75	4.23 ± 1.63^*^
	GMED:ADD	2.10 ± 1.08	3.01 ± 1.47	3.92 ± 1.08
	TA:mGCM	1.78 ± 1.04	3.06 ± 1.36	4.27 ± 0.31^**†^
Terminal swing (87-100%)	RA:ES	2.76 ± 1.20	2.17 ± 1.05	1.20 ± 0.37^*^
	RA:MF	2.49 ± 1.29	2.30 ± 1.12	1.41 ± 0.21^*^
	RF:BF	3.21 ± 1.04	3.30 ± 1.20	4.92 ± 1.20^*†^
	VMO:BF	3.35 ± 1.29	3.40 ± 1.34	4.32 ± 1.31
	GMED:ADD	1.83 ± 1.12	1.99 ± 0.87	3.00 ± 1.24^**††^
	TA:mGCM	1.84 ± 1.24	2.04 ± 1.12	4.92 ± 2.15^**††^

Values are expressed as the number or mean ± standard deviation

*RA* Rectus abdominis, *ES* Erector spinae, *MF* Multifidus, *RF* Rectus femoris, *BF* Biceps femoris, *VMO* Vastus medialis oblique, *GMED* Gluteus medius, *ADD* Adductor longus, *TA* Tibialis anterior, *mGCM* the medial part of Gastrocnemius medialis

* Difference from the young (*p* < 0.05), ** Difference from the young (*p* < 0.01), † Difference from the middle-aged (*p* < 0.05), †† Difference from the middle-aged (*p* < 0.05)

### CI in swing phase

Elderly adults had a lower trunk part CI (RA:ES) during the initial swing than young adults (P < 0.05). In the mid-swing phase, elderly adults had significantly decreased CI levels in the trunk part (RA:ES, *p* < 0.05; RA:MF, *p* < 0.05) by age. However, elderly adults had a higher thigh part CI than young adults (RF:BF, *p* < 0.01; VMO:BF, *p* < 0.05). In the terminal swing phase, elderly adults had a significantly lower trunk part CI level than young adults (*p* < 0.05). Finally, in the mid- and terminal swing phase, elderly adults had significant increase in the CI level of the shank part (TA:mGCM, *p* < 0.05) by age (Table 2).

### Relationship between CI and gait speed

**Fig. 2:**
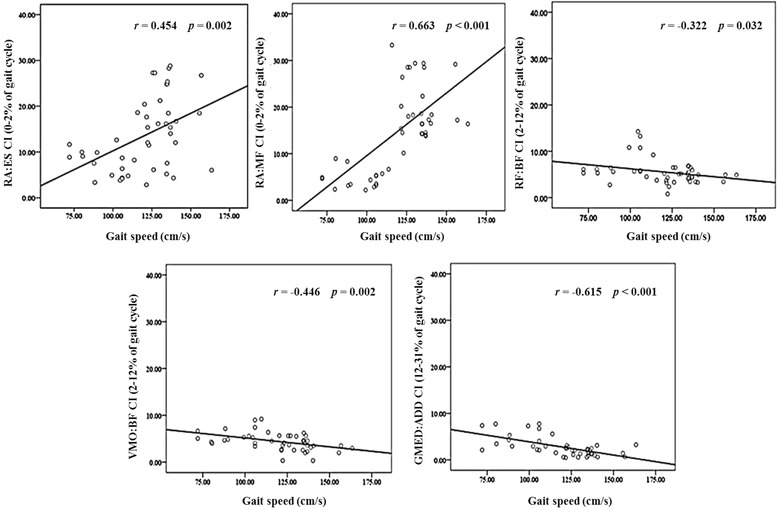
Correlation between co-activation and gait speed in stance phase. The trunk part CI revealed a significant positive partial correlation with gait speed (RA:ES, *r* = 0.454, *p* = 0.002; RA:MF, *r* = 0.663, *p* < 0.001). However, significant negative partial correlations were found between the thigh part CI and gait speed (RF:BF, *r* = -0.322, *p* = 0.032; VMO:BF, *r* = -0.446, *p* = 0.002; GMED:ADD, *r* = -0.615, *p* < 0.01). RA: Rectus abdominis, ES: Erector spinae, MF: Multifidus, RF: Rectus femoris, BF: Biceps femoris, VMO: Vastus medialis oblique, GMED: Gluteus medius, ADD: Adductor longus

**Fig. 3:**
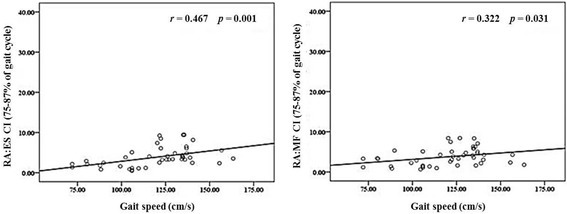
Correlation between co-activation of trunk and gait speed in swing phase. The trunk part CI showed a significant positive partial correlation with gait speed (RA:ES, *r* = 0.467, *p* = 0.001; RA:MF, *r* = 0.322, *p* < 0.031). RA: Rectus abdominis, ES: Erector spinae, MF: Multifidus

In the stance phase, the trunk part CI had a significantly positive partial correlation with gait speed (RA:ES, *r* = 0.454, *p* = 0.002; RA:MF, *r* = 0.663, *p* < 0.001). However, significant negative partial correlations were found between the thigh part CI and gait speed (RF:BF, *r* = -0.322, *p* = 0.032; VMO:BF, *r* = -0.446, *p* = 0.002; GMED:ADD, *r* = -0.615, *p* < 0.01) (Fig. 2). In the mid-swing portion of the swing phase, the trunk part CI showed a significant positive partial correlation with gait speed (RA:ES, *r* = 0.467, *p* = 0.001; RA:MF, *r* = 0.322, *p* < 0.031) (Fig. 3).

## Discussion

This study was designed to quantify the co-activation of the trunk and the lower extremity in young, middle-aged, and elderly adults during gait at a self-selected speed. It was also designed to demonstrate the relationship between muscle co-activation and gait speed. Although previous studies have demonstrated higher lower extremity muscle co-activation during gait in elderly adults compared with young adults [7, 23], no studies have quantified additional trunk co-activation during gait in the elderly. The present study focused on quantifying trunk muscle and lower extremity co-activation for stability and has made several important findings. Trunk co-activation during gait was lower in the elderly adults than in young and middle-aged adults. In contrast, lower extremity co-activation during gait was higher in elderly adults than in young and middle-aged adults. A decrease in trunk co-activation was correlated with slower gait. These results suggest that less trunk muscle co-activation in elderly adult leads to poor postural stability during gait.

Several studies identified that high levels of co-activation are associated with aging, which may impair gait performance [24, 25]. Excessive muscle co-activation in elderly adults has been described as a strategy to stiffen joints and enhance stability [7]. Increasing joint stiffness is used to compensate for the many neuro-motor impairments associated with aging [26], including reduced muscle strength, proportion of fast muscle fibers, and fear of falling [27]. In particular, a previous study found that older fallers had lower knee extensor strength, which was associated with a reduction of RF activation at the initial contact phase during gait. To compensate for this, elderly adults had higher thigh co-activation during gait [28]. Therefore, increased muscle co-activation with locomotion in elderly adults may be a necessary change to compensate for poor stability. However, excessive co-activation in elderly adults is likely to increase the energy cost of locomotion, thereby inducing fatigue and increasing the risk of falling [7]. This study results extend the co-activation data on the lower extremity and suggest that antagonist co-activation may be contribute to the age-related increase in the energy cost of locomotion.

Interestingly, we also observed a decrease in trunk co-activation by age during gait. Walter & Morris (1972) found that the trunk muscle must balance (i.e., stability) during gait on the pelvis, which moves along vertical and lateral axes [29]. Locomotion is associated with phasic activity in the rectus abdominis, the major portion of which is observed to occur before significant activity of the back muscle. The rectus abdominis appears to exert a stabilizing flexion force [30]. In addition, activity of the erector spinae and multifidus muscles stabilizes the trunk in the lateral plane [31]. Furthermore, trunk co-activation during walking requires a stability pattern of muscle forces for balance in locomotion [32]. Age-related trunk co-activation may be due in part to a greater need for active muscular stabilization of locomotion in elderly adults. However, our study results found significant poor co-activation of the trunk by aging. This observation supports the finding that elderly adults are associated with reduced postural control ability. Decreased trunk co-activation in elderly adults could contribute to an increased risk of falling due to unstable gait.

Gait speed influences co-activation of the trunk and lower extremity [7]. Our results showed higher gait speed in elderly adults than in the young and middle-aged. Furthermore, our study found a significant negative correlation between gait speed and muscle co-activation of the lower extremity. This finding suggests that elderly adults probably can utilize a significant muscle co-activation strategy when walking with decreased gait speed. In addition, co-activation of the trunk revealed a significant positive partial correlation with gait speed. This finding may suggest that less muscle co-activation of trunk is related with locomotive instability in elderly adults.

This is a pilot study with a significant small sample size to support the study's cross-sectional design. Thus, the current results warrant caution such as the small sample comparisons between the groups and the increased co-morbidity and cognitive impairment in the elderly group as compared to the middle-age and younger groups. Future research should reproduce this study results with larger samples and individuals with similar characteristics.

## Conclusions

This study asserts that there are age-related differences in the co-activation of the trunk and lower extremity muscles during gait. Elderly adults have significantly higher co-activation of lower extremity muscles than young and middle-aged adults at their preferred gait speed. In contrast, trunk co-activation is significantly lower in elderly adults than in young and middle-aged adults. Clarification of the relationship between trunk co-activation and locomotor instability will be helpful for developing optimal rehabilitation of elderly people to prevent fall.

## Abbreviations

ADD: Adductor longus
BF: Biceps femoris
EO: External oblique
ES: Erector spinae
GMAX: Gluteus maximus
GMED: Gluteus medius
RF: Rectus femoris
MF: Multifidus of the lower back
mGCM: The medial part of gastrocnemius
MVC: Maximum voluntary contraction
RA: Rectus abdominis
sEMG: Surface electromyographic analysis
SPPB: Short physical performance battery
TA: Tibialis anterior
VMO: Vastus medialis oblique.
